# 42 Multimodal NRF2 and mTOR-targeted Microparticle-based Therapy Reprograms Systemic and Pulmonary Immune Programming After Combined Injury

**DOI:** 10.1093/jbcr/irae036.042

**Published:** 2024-04-17

**Authors:** Matthew Alves, Robert Maile, Ryan Clark, Denise A Hernandez, Micah L Willis, Madelyn P Smythe, Shannon Wallet, Ben Keselowsky

**Affiliations:** University of Florida, Gainesville, Florida; University of Florida, Ocala, Florida; University of Florida, Gainesville, Florida; University of Florida, Ocala, Florida; University of Florida, Gainesville, Florida; University of Florida, Ocala, Florida; University of Florida, Gainesville, Florida; University of Florida, Ocala, Florida; University of Florida, Gainesville, Florida; University of Florida, Ocala, Florida; University of Florida, Gainesville, Florida; University of Florida, Ocala, Florida; University of Florida, Gainesville, Florida; University of Florida, Ocala, Florida; University of Florida, Gainesville, Florida; University of Florida, Ocala, Florida

## Abstract

**Introduction:**

Despite recent advancements in burn wound management, individuals afflicted with severe burn injuries face heightened susceptibility to opportunistic infections, largely attributed to a hyper pro-inflammatory response followed by a chronic compensatory anti-inflammatory response. Our prior research has identified 1) Nuclear Factor-Erythroid-2-Related Factor (NRF2) as a critical immunomodulatory component, that when activated, induces protective anti-inflammatory pathways after injury, and 2) Mammalian Target of Rapamycin (mTOR) that, when inhibited, reduces pro-inflammatory responses. However, therapeutic use of these targets is limited, as known modulators of these pathways are insoluble in saline and require long-term application. We hypothesized that administering NRF2 agonist (CDDO) in addition to Rapamycin encapsulated in soluble Poly (lactic-co-glycolic acid) (PLGA) microparticles (MP) will reduce the acute hyper-inflammatory response following a burn injury.

**Methods:**

To assess this, we conducted in vivo experiments using our murine model of burn and smoke inhalation (BI) injury. We utilized female C57BL/6 mice and separated them into four groups (n=6 per group): (1) Sham, (2) BI, (3) BI+CDDO, and (4) BI+CDDO+Rapamycin MP (combo-MP). An hour after the burn, the mice were resuscitated, and MP administered IP. After 48 hours, we collected and isolated total splenic and lung tissue mRNA and analyzed immune gene expression using nanoString.

**Results:**

We found significant changes in the activation patterns of immune genes and their associated pathways. For example, in mice administered with CDDO-MP, splenic tissue displayed a significant reduction (p < 0.05) in the activation of inflammatory genes when compared to untreated BI, such as CASP1 gene and CD9. Mice treated with combo-MP had a significant reduction in inflammatory genes associated with mTOR pathway, such as CCL9 and C3 when compared to those only treated with CDDO-MP. To examine the molecular pathways influenced by the drugs, we conducted an Ingenuity Pathway Analysis (IPA). The IPA confirmed the significantly (p-value < 0.05) increased anti-inflammatory signaling by downregulation of Pathogen Induced Cytokine Storm Signaling when compared to CDDO mice

**Conclusions:**

Our findings strongly suggest that the multi-modal MP-based therapy holds considerable promise in reprogramming the immune response after burn injuries, particularly by mitigating the hyper-inflammatory phase.

**Applicability of Research to Practice:**

This research identifies targets that are likely applicable to therapeutic targeting to reduce patient morbidity and mortality after burn and combined injuries.

